# The Emerging Role of Hepatocellular eNOS in Non-alcoholic Fatty Liver Disease Development

**DOI:** 10.3389/fphys.2020.00767

**Published:** 2020-07-03

**Authors:** Rory P. Cunningham, Ryan D. Sheldon, R. Scott Rector

**Affiliations:** ^1^Research Service, Harry S. Truman Memorial Veterans’ Hospital, Columbia, MO, United States; ^2^Department of Nutrition and Exercise Physiology, University of Missouri, Columbia, MO, United States; ^3^Metabolic and Nutritional Programming, Center for Cancer and Cell Biology, Van Andel Institute, Grand Rapids, MI, United States; ^4^Medicine-Division of Gastroenterology and Hepatology, University of Missouri, Columbia, MO, United States

**Keywords:** eNOS, nitric oxide, mitochondria, NAFLD, NASH

## Abstract

Non-alcoholic fatty liver disease (NAFLD) is comprised of a spectrum of liver injury ranging from excess fat accumulation in the liver (steatosis), to steatohepatitis (NASH), to its end stage of cirrhosis. A hallmark of NAFLD progression is the decline in function of hepatic mitochondria, although the mechanisms remain unresolved. Given the important role endothelial nitric oxide synthase (eNOS) plays in mitochondrial dynamics in other tissues, it has emerged as a potential mediator of maintaining mitochondrial function in the liver. In this mini review, we summarize the most relevant findings that extends current understanding of eNOS as a regulator of mitochondrial biogenesis, and identifies a potential additional role in mitochondrial turnover and attenuating inflammation during NAFLD development and progression.

## Introduction

Non-alcoholic fatty liver disease (NAFLD) is a progressive disease of the liver that ranges on a wide pathological spectrum from hepatic steatosis to a more severe non-alcoholic steatohepatitis (NASH) phenotype, which can progress to fibrosis and cirrhosis (Buzzetti et al., 2016). Numerous factors are involved in the progression of NAFLD, including changes in lipid metabolism, insulin resistance, inflammatory cytokines, and oxidative stress (Brunt, 2010). NAFLD has a high incidence rate, with reports in the general population ranging from 10 to 30% and as high as 80 to 100% in obesity and morbid obesity (Vernon et al., 2011; Chalasani et al., 2012). NAFLD progression is the most rapidly increasing indication for liver transplantation in the United States (Wong et al., 2015) and now can be considered a multisystem disease affecting many extra-hepatic organs. Indeed, NAFLD is an independent risk factor for cardiovascular, liver-related, and all-cause mortality (Targher et al., 2008; Stepanova et al., 2013), and doubles the risk of type 2 diabetes development (Musso et al., 2011). Currently, there are no FDA-approved pharmacological treatments for NAFLD.

### Hepatic Mitochondrial Dysfunction and NAFLD

While the mechanisms behind NAFLD development have not been fully elucidated, mounting evidence suggests that hepatic mitochondrial dysfunction is tightly linked to disease progression (Caldwell et al., 1999; Pessayre and Fromenty, 2005; Nassir and Ibdah, 2014). Characteristics of hepatic mitochondrial dysfunction in the setting of NAFLD/NASH progression include decreased electron transport chain (ETC) content, abnormal mitochondrial morphology, and reduced respiration and β-oxidation (Wei et al., 2008). However, there is some controversy surrounding this timing of hepatic mitochondrial dysfunction during NAFLD development. In rodent models, a decline in hepatic mitochondrial function is observed early in fatty liver development; our group has shown that mitochondrial dysfunction in the liver even precedes hepatic steatosis development (Rector et al., 2010). In human NAFLD/NASH development it is not as clear – previous studies have reported either a compensatory increase or no change in hepatic mitochondrial function and TCA cycle flux during fatty liver development in humans (Sunny et al., 2011; Petersen et al., 2016; Fletcher et al., 2019), but this is lost during NASH progression (Morris et al., 2011; Koliaki et al., 2015). Moreover, NASH patients present with porous mitochondria and elevated hepatic oxidative stress, along with impaired mitochondrial biogenesis and decreased ETC complexes (Koliaki et al., 2015). NAFLD is also associated with increased mitochondrial membrane potential, electron leak, and elevated ROS production (Rolo et al., 2012). In fact, NASH patients have lower maximal hepatic mitochondrial respiration and increased proton leakage, despite having higher mitochondrial mass, and this is associated with elevated hepatic oxidative stress, greater H_2_O_2_ emission, and reduced anti-oxidant capacity (Koliaki et al., 2015). Despite the debate over its timing during NAFLD/NASH development, mitochondrial dysfunction is clearly implicated in exacerbating disease progression, and therapies that target hepatic mitochondria may provide novel avenues for NAFLD/NASH treatment. However, the mechanisms behind hepatic mitochondrial function, and ultimately NAFLD development are unresolved. In this review, we posit that one potential mediator of hepatic mitochondrial dysfunction and NAFLD progression is the loss of endothelial nitric oxide synthase (eNOS), and its product nitric oxide (NO), due to this enzyme’s well-established role in metabolic health and mitochondrial dynamics (discussed later). We will focus on the roles of hepatocellular eNOS and NO in maintaining a healthy mitochondrial pool within the liver and how this may pertain to NAFLD pathogenesis.

### Nitric Oxide

Since the discovery of a role for NO in maintaining cardiovascular homeostasis by Furchgott, Ignarro, and Murad, for which they shared a Nobel Prize in Physiology or Medicine in 1998, numerous physiological and pathophysiological functions of NO have been described. NO is an autocrine and paracrine signaling gas that has many diverse functions and molecular targets, such as regulating neurotransmission, vascular tone, gene transcription, mRNA translation, and post-translational modification [reviewed in detail (Martínez-Ruiz et al., 2011; Förstermann and Sessa, 2012)]. NO is produced by the nitric oxide synthase (NOS) enzyme, which consists of three isoforms; neuronal NOS (nNOS), inducible NOS (iNOS), and endothelial NOS (eNOS). All three isoforms catalyze the production of NO by converting L-arginine to L-citrulline, with oxygen, reduced nicotinamide-adenine-dinucleotide phosphate (NADPH), and tetrahydrobiopterin (BH4) used as essential substrates and cofactors (Bendall et al., 2005). The biological role of NO is often concentration dependent; pathological levels (μM) are typically produced by iNOS as an immune defense mechanism when macrophages are induced leading to cytotoxicity. Alternatively, physiological levels of NO (nM) are constitutively produced by nNOS and eNOS. The effects of NO are largely concentration dependent, and as the concentration of a gas reduces by a decay constant as the distance from its point of origin, the enzymes that produce NO are poised to be a robust cellular tool in tailoring subcellular responses as needed. This is most notably apparent in the well characterized role of eNOS in vascular endothelial cells, where it is located at the luminal plasma membrane in order to rapidly increase its production of NO and cause vasodilation in response to increases in shear stress. Since its initial discovery as a potent vasodilator in endothelial cells, eNOS has been shown to provide important regulatory functions in a variety of tissues.

### eNOS Activation

**FIGURE 1:**
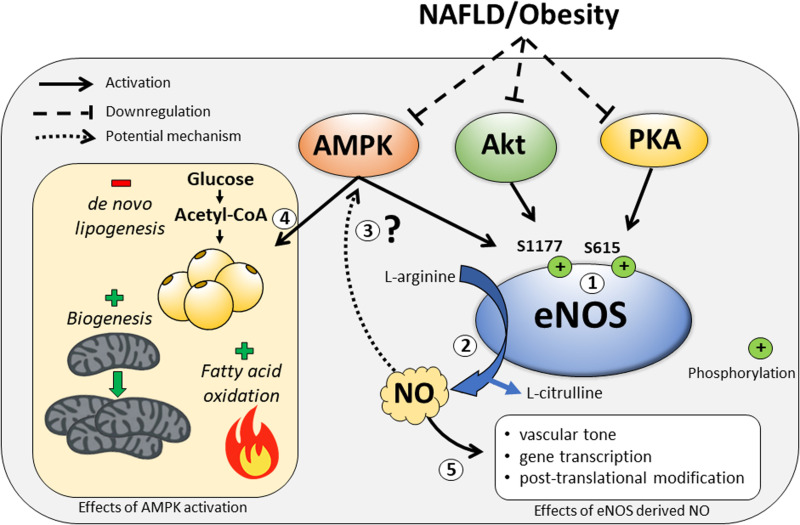
The regulation of eNOS by the kinases AMP-activated protein kinase (AMPK), serine/threonine protein kinase (Akt), and protein kinase A (PKA), and some of the downstream metabolic consequences of eNOS modulation by these signals. (1) In response to external and internal stimuli, eNOS is activated by the kinases AMPK, Akt, and PKA at the major phosphorylation sites – S1177 and S615. These kinases can also be downregulated during NAFLD/obesity. (2) Upon eNOS activation, nitric oxide (NO) is produced as a byproduct during the conversion of L-arginine to L-citrulline. (3) It is unknown whether eNOS-derived NO is required for the metabolic benefits of AMPK activation. (4) Activation of AMPK results in decreased *de novo* lipogenesis and increased fatty acid oxidation and mitochondrial biogenesis. (5) Other downstream effects of eNOS-derived NO upon activation of eNOS.

Activity of eNOS is largely controlled by phosphorylation at multiple sites that can positively or negatively influence its activity. Seven phosphorylation sites have been characterized, including two stimulatory sites (S615, S1177), three inhibitory sites (S114, T495, Y657), and two sites for which the influence on eNOS activity is undetermined (Y81, S633) (Bauer et al., 2003; Mount et al., 2007). Of particular interest is that eNOS is activated via S1177 and S615 phosphorylation by AMP-activated protein kinase (AMPK) (Chen et al., 1999), serine/threonine protein kinase (Akt) (Fulton et al., 1999), and protein kinase A (PKA) (Michell et al., 2002). These data highlight the ability for eNOS activity to be modulated both by external stimuli (Akt, PKA) as well as the internal metabolic state (AMPK) of the cell. Many NAFLD therapeutics target AMPK activation due its purported benefits on *de novo* lipogenesis inhibition, increased fatty acid oxidation, and stimulation of mitochondrial biogenesis (Smith et al., 2016). Interestingly, activation of AMPK by exercise (Lee-Young et al., 2010) or metformin (Zou et al., 2004) does not occur in eNOS knockout (KO) mice; whereas, NO donors can stimulate AMPK phosphorylation (Zhang et al., 2008). This lends support to the notion that eNOS may exert its metabolic benefits in an AMPK-dependent manner and play an important role in scenarios where metabolism and/or energy state is perturbed, i.e., obesity/NAFLD (Figure 1).

### The Link Between eNOS and NAFLD

Mounting evidence from whole body eNOS KO models demonstrate its importance for metabolic health. Mice lacking eNOS have elevated FFAs and triglycerides, as well as reduced mitochondrial content and beta-oxidation in skeletal muscle (Le Gouill et al., 2007). Additionally, eNOS KO mice develop insulin resistance at the level of the liver and other peripheral tissues (Shankar et al., 2000), and accumulate more liver fat compared to WT mice on a high-fat diet (Nozaki et al., 2015). Taken together, these data clearly outline an important role for eNOS in metabolic health. In the context of NAFLD development, the vast majority of studies examining eNOS have focused on its role in regulation of hepatic blood flow and vascular resistance. Portal hypertension is a major complication in liver disease caused by increased intrahepatic vascular resistance mediated in part by a reduction in eNOS derived NO (Iwakiri and Groszmann, 2007); cirrhotic livers also present with significantly reduced hepatic eNOS activity (Rockey and Chung, 1998; Shah et al., 1999), while NAFLD patients across varying degrees of severity show marked hepatic eNOS dysfunction (Persico et al., 2017).

There is also limited evidence indicating that loss of eNOS is associated with increased hepatic steatosis. Double KO mice obtained from crossing obese leptin receptor deficient (*Leprdb/db*) mice crossed with eNOS KO mice exhibit elevated hepatic steatosis compared with *Leprdb/db* (Mohan et al., 2008). Similarly, eNOS KO mice fed a 60% high fat diet for 12-weeks accumulate ∼25% more TAG than WT counterparts, a finding which the authors attributed to attenuated microsomal triglyceride transfer protein (TAG export) activity in eNOS KO mice (Nozaki et al., 2015). Finally, hepatic TAG accumulation was increased with 4 weeks of NOS inhibition with the non-selective NOS inhibitor (N^ω^ -nitro-L-arginine methyl ester; L-NAME), in hyperphagic OLETF rats (Sheldon et al., 2015). Moreover, we also have shown that reduced eNOS activation is associated with NAFLD progression (Sheldon et al., 2014, 2019). Importantly, loss of eNOS activation, as well as NAFLD development, were prevented with chronic voluntary wheel running exercise, indicating a potential role for exercise-induced elevations in eNOS activity in liver health (Sheldon et al., 2014). The whole body eNOS null model coupled with evidence of reduced eNOS activation with NAFLD development demonstrates a potential protective role for eNOS in the pathogenesis of NAFLD (Table 1). However, these studies fail to define the role of hepatocyte-specific eNOS in this pathogenesis, as this enzyme is present in other cell types within the liver.

**TABLE 1:** Manipulations of eNOS and their effects on NAFLD and mitochondrial outcomes in liver and other tissues.

Authors	Model	Comments/outcomes
Le Gouill et al., 2007	Whole body eNOS KO	↓ Mitochondrial content and β-oxidation in skeletal muscle vs. WT mice
Shankar et al., 2000	Whole body eNOS KO	↑ Hepatic insulin resistance vs. WT mice
Nozaki et al., 2015	Whole body eNOS KO	↑ Hepatic steatosis, ↓ TAG export and hepatic blood flow vs. WT mice
Mohan et al., 2008	Double KO mice – leptin receptor and eNOS KO	↑ Hepatic steatosis vs. leptin KO only mice
Sheldon et al., 2015	NOS inhibition via L-NAME	↑ Hepatic steatosis and stellate cell activation, ↓ hepatic mitochondrial function vs. OLETF controls
Sansbury et al., 2012	Global eNOS overexpression	↓ Diet-induced obesity, ↑ mitochondrial function in adipose tissue vs. WT mice
Trevellin et al., 2014	Whole body eNOS KO	↓ Exercise-induced mitochondrial biogenesis and content in adipose tissue vs. WT mice
Vettor et al., 2014	Whole body eNOS KO	↓ Exercise-induced mitochondrial biogenesis and content in cardiac tissue vs. WT mice
Sheldon et al., 2019	eNOS KO hepatocytes	↓ Mitochondrial biogenesis, mitophagy, and fatty acid oxidation vs. control hepatocytes
Seth et al., 2015	Systemic NO donor	↓ NASH progression via lower M1 macrophage polarization vs. WT mice
Langer et al., 2008	Systemic NO donor	↓ NASH progression via increased hepatic stellate cell apoptosis
Liu et al., 2003	Liver-specific NO donor	↓ Drug-induced hepatic oxidative stress and DNA damage vs. untreated control mice
Moal et al., 2006	Liver-specific NO donor	↓ Bile duct ligation-induced hepatic fibrosis vs. placebo treated rats
Maslak et al., 2015	Liver-specific NO donor	↓ Hepatic steatosis and DNL vs. untreated control mice

*↑, increased; ↓, decreased; KO, knockout; NOS, nitric oxide synthase; NO, nitric oxide; WT, wild-type; TAG, triglyceride; vs, versus; DNL, *de novo* lipogenesis.*

### Hepatocellular eNOS

Considerable discrepancy exists in the literature regarding the distribution of eNOS in the liver. In a seminal report, Shah et al. (1997) demonstrated in rats that eNOS regulates sinusoidal perfusion and immunohistochemistry demonstrated its expression was restricted to liver endothelial cells. Based largely on this report, numerous subsequent studies on hepatic eNOS presume a mere endothelial localization of eNOS yet offer little, if any, supporting evidence (Shah et al., 1997, 1999, 2001; Pasarín et al., 2012). However, mounting evidence identifies a more ubiquitous eNOS distribution in the liver; McNaughton et al. (2002) used immunohistochemistry and *in situ* hybridization to demonstrate widespread eNOS expression in human liver samples. In addition, Mei and Thevananther (2011), demonstrated an increase in eNOS S1177 phosphorylation in isolated primary hepatocytes following epidermal growth factor stimulation. Interestingly, eNOS was localized in the cytosol in a pattern consistent with hepatocyte mitochondrial distribution. Similarly, increased eNOS activation in isolated primary wild-type hepatocytes was observed with BH4 administration (Abudukadier et al., 2013).

In one of the most comprehensive proteomic analysis of any organ to date, Azimifar et al. (2014) used density gradient centrifugation and magnetic affinity cell sorting techniques to obtain highly purified murine hepatic cell types; hepatocytes, Kupffer cells, liver sinusoidal endothelial cells, hepatic stellate cells, and intrahepatic cholangiocytes. Using an advanced liquid chromatography tandem mass spectrometry approach, they clearly identified eNOS in their purified hepatocytes, with its highest expression in Kupffer cells and liver sinusoidal endothelial cells. Using the same methodology, our group has confirmed these findings by identifying eNOS expression in isolated primary hepatocytes from WT mice using magnetic-activated cell sorting and immunofluorescence (Sheldon et al., 2019). Taken together, these more recent and methodologically robust data strongly imply the presence of eNOS in hepatocytes.

### Mitochondrial NOS

As a further confounder to the role of the NOS enzymes in hepatocytes, there is a body of evidence supporting the existence of mitochondrial NOS (mtNOS) that is present on the inner mitochondrial membrane. As there is no fourth NOS gene in mammalian genomes, mtNOS is thought to be a variant of one of the three known NOS isoforms. Initial reports identified eNOS as the mtNOS in skeletal muscle, brain, and liver (Bates et al., 1995; Kobzik et al., 1995). However, it was later demonstrated that eNOS physically associates with the outer leaflet of the outer mitochondrial membrane (Gao et al., 2004) and as such is not believed to be the “bona fide” mtNOS on the inner membrane (Ghafourifar and Cadenas, 2005). Instead, evidence exists to support both nNOS and iNOS as mtNOS. Persichini et al. (2005) demonstrated that the PDZ domain nNOS physically interacts with cytochrome c oxidase (COX) and used immunogold to identify nNOS in the inner mitochondrial membrane. Alternatively, Tatoyan and Giulivi (1998) identified mtNOS as iNOS in purified rat liver mitochondria. Regardless of isoform or sub-mitochondrial localization, it is apparent that a NOS isoform is present in hepatic mitochondria and, given the current discussion, this may have significant implications in hepatic mitochondrial physiology. The influence and significance of “mtNOS” as it relates to NAFLD has not previously been addressed and is outside the scope of this mini review.

## The Role of eNOS in Mitochondrial Dynamics and Function

### eNOS and Mitochondrial Respiration

How eNOS plays a role in NAFLD pathogenesis may be its function as a mediator of mitochondrial function. A key physiological role of eNOS (and other NOS enzymes) derived NO is its well-characterized interaction with COX in the mitochondrial ETC. NO inhibits COX via both competitive and non-competitive sites (Mason et al., 2006). Importantly, these inhibitory actions of NO on COX were demonstrated across both pathological and physiological spectrum of both NO and O_2_ concentrations. These data indicate that the rate of O_2_ consumption at a given [O_2_] is inversely proportional to [NO]. In addition, NO may influence other aspects of mitochondrial bioenergetics, including attenuation of complex I and III, which are known sites for ROS production through S-nitrosylation (Clementi et al., 1998; Brown and Borutaite, 2004), and NO-dependent inhibition of the TCA cycle in rat hepatocytes (Stadler et al., 1991). Thus, constitutive NO production may act to adjust mitochondrial respiration, in a manner similar to metformin – which exerts its hepatic benefits by inhibiting mitochondrial respiration to increase hepatic AMPK activation (Lin et al., 2000). Taking hypoxia, a known activator of eNOS, as an example, such respiratory inhibition by NO would prevent excess O_2_ consumption by mitochondria closest to the O_2_ source and allow for more ubiquitous tissue O_2_ distribution. Similarly, in a fed condition, which activates eNOS via insulin mediated Akt activation, this NO throttle could reduce substrate oxidation in the most proximal mitochondria thus limiting the extent of ROS production in this microdomain. This function may be particularly relevant to the attenuation of mitochondrial derived ROS in NAFLD by attenuating electron transport flux and membrane potential in the face of increased ETC pressure from nutrient excess.

### eNOS and Mitochondrial Biogenesis

The link between eNOS derived NO and its regulation of mitochondrial function in other tissues is well established. In a seminal paper by Nisoli et al. (2003) the addition of an NO donor to cultured brown adipocytes induced markers of mitochondrial biogenesis and content in a cyclic GMP and peroxisome proliferator receptor γ coactivator α (PGC1α) dependent manner. In the same study, cold exposure-induced mitochondrial biogenesis in brown adipose tissue was significantly attenuated in whole body eNOS null mice, while eNOS null mice also presented with reduced markers of mitochondrial content in brain, heart, and liver tissue (Nisoli et al., 2003). Furthermore, calorie restricted-induced mitochondrial biogenesis is severely attenuated in eNOS null mice (Nisoli et al., 2005). The reverse is also true – global eNOS overexpression prevented diet induced obesity while increasing markers of mitochondrial biogenesis and activity in adipose tissue (Sansbury et al., 2012). Additionally, TNFα-induced downregulation of eNOS expression impairs mitochondrial biogenesis and function in different tissues of obese rodents (Valerio et al., 2006), further solidifying the link between eNOS and mitochondrial biogenesis. Interestingly, exercise-induced adaptations in mitochondrial health may be dependent on eNOS. In fact, 6 weeks of swim training increased mitochondrial biogenesis, mitochondrial DNA content, and glucose uptake in subcutaneous adipose tissue of wild-type but not eNOS KO mice (Trevellin et al., 2014). Similarly, genetic ablation of eNOS attenuated exercise-induced increases in mitochondrial biogenesis and function in cardiomyocytes (Vettor et al., 2014). Collectively, these studies demonstrate a strong tie between eNOS and mitochondrial biogenesis (Table 1), and that exercise promotes eNOS dependent improvements in mitochondrial biogenesis and function.

### eNOS and Autophagy/Mitophagy

While there is clear evidence for a role of eNOS in the process of mitochondrial biogenesis, less is known about the ability of eNOS to regulate mitochondrial turnover. This turnover hinges on the intimately linked processes of biogenesis and autophagy – where dysfunctional, aberrant mitochondria are removed via selected autophagy (mitophagy) and replaced in an effort to maintain mitochondrial homeostasis within the cell (Kim et al., 2007). Indeed, impaired mitophagy in the liver leads to accumulation of dysfunctional mitochondria, increased oxidative stress, and elevated steatosis (Dorn, 2010). BNIP3 (BCL2/adenovirus E1B 19 kDa protein-interacting protein 3) is a cytosolic protein that docks to the outer mitochondrial membrane where it tags damaged mitochondria for autophagosomal engulfment and lysosomal degradation. Despite dramatically elevated hepatic mitochondrial mass in BNIP3 null mice, a large percentage of the mitochondria are damaged (have no membrane potential) and have defective mitochondrial β-oxidation capacity, and the animals develop hepatic steatosis (Glick et al., 2012).

**FIGURE 2:**
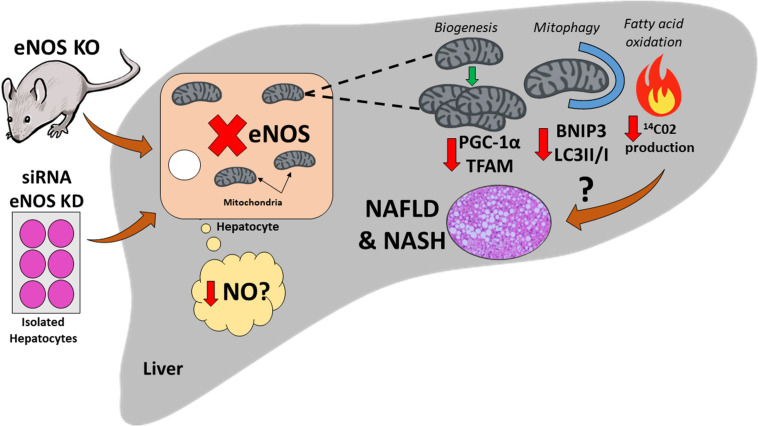
Schematic illustration of the potential role that hepatocellular endothelial nitric oxide synthase (eNOS) may play in non-alcoholic fatty liver disease (NAFLD) development. Genetic ablation of eNOS in mice, as well as small interfering RNA (siRNA) knockdown (KD) of eNOS in isolated primary hepatocytes result in hepatocytes lacking eNOS. This leads to a reduction in markers of hepatic mitochondrial biogenesis [PPARγ coactivator-1α (PGC-1 α), mitochondrial transcription factor A (TFAM)], and also markers of autophagy/mitophagy [BCL-2-interacting protein-3 (BNIP3), 1A/1B light chain 3B (LC3)], and decreased fatty acid oxidation in primary hepatocytes [as measured by complete ^14^Co_2_ production (Sheldon et al., 2019)]. Whether this process is governed by a reduction in nitric oxide (NO) is yet to be determined. This impairment in hepatic mitochondrial dynamics can lead to mitochondrial dysfunction, and ultimately may cause/exacerbate NAFLD development.

The data on eNOS and autophagy/mitophagy are limited; however, there is strong evidence for NO regulation of autophagy. NO has consistently shown to be a potent activator of AMPK (Zhang et al., 2008). This directly promotes autophagy/mitophagy by the AMPK-induced activation of Unc-51 Like Autophagy Activating Kinase 1 (Ulk1) at Ser 317 and Ser 777 (Kim et al., 2011) – a key initiating step in the autophagosome formation. Indeed, NO stimulates autophagy in breast cancer cells by suppression of mTORC1 (Ulk1 inhibitor) and activation of AMPK (Tripathi et al., 2013). This resulted in elevated light chain 3 (LC3) II/I ratio and decreased p62 levels, indicative of increased autophagosome formation and ultimately its targeted degradation. Recent work from our lab has demonstrated hepatocellular eNOS as a novel regulator of mitochondrial homeostasis and maintaining mitophagic capacity (Sheldon et al., 2019). Hepatic mitochondria from eNOS null mice displayed decreased markers of mitochondrial biogenesis (PGC1α, TFAM) and autophagy/mitophagy (BNIP3, LC3II/I). In addition, *in vitro* siRNA-induced knockdown of eNOS in primary hepatocytes reduced fatty acid oxidation and impaired the induction of BNIP3 upon mitophagic stimulation. Collectively, these data demonstrate a novel role for hepatocellular eNOS in maintaining a healthy mitochondrial pool within the hepatocyte and may contribute to curbing NAFLD development (Figure 2). Further *in vivo* manipulation of hepatocellular eNOS is required to tease out its significance in hepatic mitochondrial dynamics and NAFLD development.

### eNOS and Advanced Liver Disease

Our lab has recently demonstrated that normal hepatic eNOS activation is dramatically reduced in the obese OLETF rat during the transition from hepatic steatosis to NASH (Sheldon et al., 2014), implicating the loss of normal eNOS function in the advancement of liver disease. In a follow-up study, treating obese OLETF rats with L-NAME for 4 weeks caused reduced hepatic mitochondrial respiration, increased hepatic TAG accumulation, and induced a more NASH-like phenotype (increased stellate cell and Kupffer cell activation markers) compared with untreated obese controls (Sheldon et al., 2015). These studies support previous work implicating reduced eNOS activity and NO production in the activation of Kupffer cells and hepatic stellate cells (Tateya et al., 2011; Xie et al., 2012).

On the other hand, increasing NO bioavailability may provide some hepatoprotection against metabolic insults. Giving a systemic NO donor has recently been shown to attenuate NASH progression, in part through lowering M1 macrophage polarization (Seth et al., 2015) and promotion of hepatic stellate cell apoptosis (Langer et al., 2008). Perhaps more encouraging, initial studies with a liver-selective NO donor, V-PVRRO/NO, which avoids the potential confounding factors of systemic NO delivery, including hypotension, have shown protection against drug induced (Liu et al., 2003) and bile duct ligation induced liver injury (Moal et al., 2006) and also reduced high fat diet induced hepatic steatosis by >50% (Maslak et al., 2015). Targeting eNOS may also be a strategy for attenuating NASH development; mice treated with relaxin-2 increased hepatic eNOS activation and attenuated Kupffer cell and stellate cell activation in methionine–choline-deficient (MCD) diet-induced NASH (Lee et al., 2019). Collectively, these data demonstrate an association between loss of hepatic eNOS function and NASH. Importantly, increasing hepatic eNOS and NO shows promise as a potential therapeutic to curb advanced liver disease progression, possibly by attenuating hepatic inflammation (Table 1). Future studies overexpressing hepatocellular eNOS in the face of a liver insult would help identify its role in mitigating NASH development.

## Conclusion

Significant strides have been made in recent years in teasing out the molecular mechanisms of NAFLD development and progression, including the pathological role of hepatic mitochondrial dysfunction. Given the well characterized role of eNOS in mitochondrial dynamics in an array of tissues, including exercise-induced mitochondrial adaptations, one could speculate that eNOS is also functioning to maintain a healthy mitochondrial pool in hepatocytes (Figure 1). Indeed, data from our group have identified a novel role for hepatocellular eNOS in NAFLD development and furthers our understanding of the molecular mechanisms involved in NAFLD pathogenesis (Figure 2). Future studies should seek to further elucidate the role of hepatocellular eNOS via direct manipulation, i.e., *in vivo* hepatocyte-specific gain and loss of function studies, and whether hepatocellular eNOS is required for hepatic mitochondrial adaptations to exercise. Uncovering a role for hepatocellular eNOS may improve our knowledge of NAFLD pathogenesis, but more importantly, also may provide a potential therapeutic target to attenuate hepatic mitochondrial dysfunction and curb NAFLD development.

## Author Contributions

RC and RS drafted the manuscript. RC, RS, and RR edited and revised the manuscript. All authors contributed to the article and approved the submitted version.

## Conflict of Interest

The authors declare that the research was conducted in the absence of any commercial or financial relationships that could be construed as a potential conflict of interest.
